# Editorial: Low threshold interventions and preventive approaches in child mental health care

**DOI:** 10.3389/frcha.2023.1286421

**Published:** 2023-11-28

**Authors:** Eva Möhler

**Affiliations:** ^1^ Saarland University, Saarbrucken, Germany; ^2^ Saarland University Hospital, Homburg, Germany; ^3^ SHG Clinic for Child and Adolescent Psychiatry, Saarbrucken, Germany

**Keywords:** adolescents, children, intervention-behavioral, adolescence, innovation

**Editorial on the Research Topic** Low threshold interventions and preventive approaches in child mental health care

Child mental health care worldwide is facing considerable challenges because of the increasing prevalence of anxiety, depression, and eating disorders in children and adolescents, specifically the increase since the pandemic (1–3). An increase in externalizing symptoms, such as aggression and impulsivity, has also been reported (3, 4). Recent studies have shown that clinically relevant mental health issues are present in about 30% of children and adolescents (5). These numbers by far exceed the capacity of medical care systems in many countries, resulting in long waiting times and increases in emergency admissions to psychiatric units (6). Because mental health issues in childhood are associated with an elevated risk of adult mental and physical disorders (7), this situation is a significant public health concern. Therefore, interventions and preventive efforts are needed that target child and adolescent mental health beyond the regular clinical system.

The goal of this Research Topic on low-threshold interventions was to invite novel and creative approaches targeting this critical situation with potential innovative solutions. Specifically, we invited evaluation studies on treatment options that can be made easily available to children and adolescents or reduce cognitive, motivational, or financial barriers to treatment.

This Research Topic presents tailored intervention studies that contribute to better equipment with psychotherapeutic tools in the field of mental health care for children. Specifically, with evaluation data on an innovative school-based intervention targeting bullying and other important issues, Wang et al. provide incentives for mental health professionals to offer preventive support before children become child and adolescent psychiatric patients. The structured grit training described in this article is a promising school-based tool to lower children's general vulnerability, lessening their likelihood of becoming a victim of bullying. The detrimental effects of bullying have been shown, for example, by Moore et al. (8); therefore, the approach presented here seems to be of value and importance for the protection of general child mental health. Moreover, interventions improving attachment are presented in this Research Topic as parent–child attachment is an integral factor for children's emotional safety and social competences (9) and, thus, a predictor of longitudinal emotional development. This informative overview demonstrates the differential benefits of different approaches, revealing desirable and stable effects of early intervention focusing on long-term child mental health and emphasizing the importance of this comprehensive review article by Hutchings et al.

Another approach, the Stress–Arousal–Regulation–Treatment, is a short intervention program (10) derived from dialectical behavioral therapy (DBT) and based on emotional awareness and skills training. It has demonstrated an improvement in emotional regulation in severely unstable adolescents in acute suicidal or impulsive crises. In this vulnerable population, commitment issues are often challenging for clinicians as patients in acute psychiatric emergency presentations and situations tend to be difficult to motivate for a longer term or cognitively challenging psychotherapeutic treatment. Therefore, this low-threshold intervention evaluated by Dixius et al. is significantly shorter than DBT and has less cognitive or motivation requirements. The increase in adaptive emotion regulation strategies and the reduction in maladaptive strategies such as self-harm, drug abuse, and suicidal behavior reported in this article have been described (11) as significant factors increasing stress resilience and supporting longitudinal emotional stability with potentially reduced mental health care needs in adulthood. According to the results of Moffitt et al. (12), interventions improving self-control may contribute to a longitudinal increase in public health by reducing unhealthy regulation strategies such as overeating, smoking or drinking, wealth, and public safety.

An approach tailoring video consultation systems to the demands of young people seems to be beneficial, as reported here by Gormley et al. Specifically, this consultation system reduces barriers to treatment, such as transportation issues, and meets adolescents in their preference of digital media, as described by Paulus et al. (13). The results presented in this study are encouraging and should encourage researchers and clinicians to engage in future studies on these promising intervention tools.

This Research Topic highlights several promising preventive efforts and low-threshold interventions that are needed in the field of child and adolescent psychiatry and psychology.
